# Vitamin D, calcium, phosphates, and magnesium serum level and bone mineral density in patients with type 1 diabetes

**DOI:** 10.3389/abp.2026.15388

**Published:** 2026-02-02

**Authors:** Agnieszka Zawada, Michał Michalak, Dariusz Naskręt, Agata Grzelka–Woźniak, Alicja Ewa Ratajczak-Pawłowska, Anna Maria Rychter, Kinga Skoracka, Aleksandra Szymczak-Tomczak, Dorota Zozulińska-Ziółkiewicz, Agnieszka Dobrowolska, Iwona Krela-Kaźmierczak

**Affiliations:** 1 Department of Gastroenterology, Dietetics and Internal Diseases, Poznan University of Medical Sciences, Poznan, Poland; 2 Department of Computer Science and Statistics, Poznan University of Medical Sciences, Poznan, Poland; 3 Department of Internal Medicine and Diabetology, Poznan University of Medical Sciences, Poznan, Poland; 4 Doctoral School, Poznan University of Medical Sciences, Poznan, Poland; 5 Laboratory of Nutrigenetics, Department of Gastroenterology, Dietetics and Internal Diseases, Poznan University of Medical Sciences, Poznan, Poland

**Keywords:** bone, calcium, diabetes mellitus, osteoporosis, vitamin D

## Abstract

**Introduction:**

Abnormal glucose metabolism, which is a common condition in patients with type 1 diabetes (T1DM), triggers a number of changes in various organs. Additionally, elevated blood glucose level also affects the bones. Bone mineral density (BMD), vitamin D, calcium, phosphates, and magnesium serum concentrations are significant factors assessed in bone metabolism. In this study, we evaluated these factors and the impact of vitamin D supplementation on vitamin D and BMD levels in T1DM patients.

**Materials and methods:**

The study included 66 adults with T1DM and a control group of 66 healthy adults of the same age and weight. Densitometric measurements of the lumbar spine (L1–L4) and femoral neck (FN) were performed using dual-energy X-ray absorptiometry. The concentration of vitamin D, calcium, phosphates, and magnesium in the blood was assessed. All patients completed a questionnaire regarding vitamin D supplementation and symptoms associated with osteoporosis.

**Results:**

Significant differences in the range, BMD, Z-score and T-score for FN and L1-L4 were observed in diabetic and non-diabetic subjects. Only 50% of participants with diabetes and osteopenia and only 40% of diabetic patients without osteopenia showed optimal vitamin D concentration. There were no differences in BMD, T-score and Z-score of FN, as well as in L1-L4 between the subjects who supplemented and those who did not supplement vitamin D.

**Conclusion:**

Patients with T1DM are more at risk of developing osteoporosis than healthy individuals. Vitamin D may not be the only factor affecting BMD. Patients with T1DM should be screened for osteoporosis and other complications. Patients with type 1 diabetes should receive higher doses of vitamin D than healthy adults and control their calcium, magnesium and phosphates serum concentrations.

## Introduction

**FIGURE 1:**
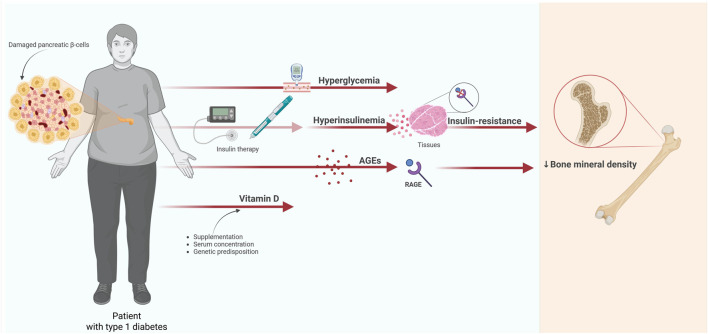
Factors influencing bone mineral density in patients with type 1 diabetes.

Type 1 diabetes mellitus (T1DM) results from an autoimmune process, which causes the damage of pancreatic β-cells and a lack of insulin secretion. This type of diabetes affects approximately 5%–7% of the diabetic population, and primarily involves young people and children. In spite of the fact that it appears relatively early and impacts a person’s entire life, it is impossible to predict who will develop this disease, and treatment is carried out throughout the patient’s life. Thus, it is crucial to introduce appropriate management and prevent these patients from developing chronic complications. Without proper treatment, the disease may result in disability, severe chronic complications, and a significant reduction in patients’ standard of living at an early age. It is essential to bear in mind that chronic hyperglycemia affects numerous organs and systems, although the most common consequences entail the nervous, cardiovascular, urinary, and visual systems. However, there are various complications where the association with hyperglycemia is poorly understood, which–in turn - may influence patients’ health. One such complication is osteoporosis. Studies indicate that the risk of hip fracture is 2.4–7 times higher in individuals with T1DM (Starup-Linde et al., 2019). Bone mineral disturbances in diabetic patients may have clinically significant consequences, such as bone fractures during a fall, or dangerous falls during a hypoglycemic episode. Proper prevention, diagnosis and early treatment of this disease can bring significant benefits to patients suffering from diabetes and significantly reduce the risk of developing disability. Furthermore, vitamin D also not only plays a pivotal role in bone mineral metabolism, but also may decrease other complications, e.g., cardiovascular diseases. The pathogenesis of osteoporosis in diabetic individuals is multifactorial. Data show that bone metabolism is affected not only by hyperglycemia, but also by increased oxidative stress, accumulation of protein glycation end products, hyperinsulinemia and insulin resistance, as well as genetic predisposition and vitamin D3 deficiency. The issues addressed in the current paper are presented in Figure 1. It is of note that vitamin D affects calcium and phosphate metabolism, as well as the level of parathyroid hormone and vitamin D receptors in bone tissue. Therefore, the assessment of mineral and vitamin D parameters should be introduced into the standard of care for diabetic patients. In addition, individuals who develop type 1 diabetes as an autoimmune disease may more frequently experience malabsorption. Nevertheless, in our study group, subjects with type 1 diabetes and coexisting celiac disease were excluded from the study. The assessment of vitamin D concentration is even more critical since patients suffering from osteoporosis or osteopenia present with lower serum vitamin D concentrations than healthy adults. However, the incidence of osteoporosis varies depending on the type of diabetes and the presence of chronic complications, although it is most commonly observed in patients with type 1 diabetes. In turn, patients with type 2 diabetes often suffer from obesity, which may have an ambiguous effect on bone mass. In the current study, we focused on a homogeneous group of patients with type 1 diabetes in order to avoid the influence of other comorbidities. Thus, we carefully selected a homogeneous group in terms of duration of diabetes, body weight, and without concomitant chronic diseases and complications that could affect bone and mineral density. The study assessed the incidence of osteoporosis and osteopenia, as well as the relationship between bone mineral density (BMD), vitamin D, calcium, phosphates, and magnesium serum concentrations in patients with type 1 diabetes. In addition, the effect of vitamin D supplementation on vitamin D and BMD levels was evaluated.

The aim of the study was to assess 25 (OH)vitamin D, calcium, phosphates, and magnesium serum concentrations and bone mineral density in patients with type 1 diabetes and to compare the results between the patients and the control group.

## Materials and methods

### Participants

The study included a group of 132 participants comprising patients with type 1 diabetes (n = 66) aged 39 ± 8 and the control group of (n = 66) healthy individuals aged 37 ± 8 years. The study group was recruited at the Metabolic Clinic of the University Clinical Hospital in Poznan and the Department of Internal Medicine and Diabetology at the Franciszek Raszeja Municipal Hospital. The control group was obtained from healthy individuals matched for age, gender, WHR, body weight and creatinine level. The number of controls per case was 1:1. The participants in both groups underwent densitometry tests and had blood tests performed at the University Clinical Hospital in Poznan. The diabetes group and control group were enrolled between September 2021 and December 2022. Recruitment was conducted until the estimated number of subjects was obtained for each endpoint. The inclusion criteria involved: type 1 diabetes (diagnosis confirmed by the presence of autoantibodies: ICA, IA2 and GAD) treated with intensive functional insulin therapy or using a personal insulin pump, age >18 years <50 years, Caucasian, diabetes duration over 5 years, consent to participate in the study. Exclusion criteria comprised: types of diabetes other than type 1, diabetes remission, post-menopausal women, presence of chronic diabetic kidney disease with GFR <60, individuals with previously diagnosed osteoporosis, as well as those treated chronically with steroids or other drugs affecting the bone mineral economy, pregnancy and other diseases that may affect bone mineral density, such as decompensated hypothyroidism or hyperthyroidism and rheumatic diseases. People who reported consuming more than 3 standard doses of alcohol per week were also not included in the study.

### Bone mineral density test

Densitometric measurements were conducted of the femoral neck (FN) and the lumbar spine (L1–L4) the using dual-energy X-ray absorptiometry with Lunar DPX-Plus (Lunar, Inc., Madison, Wisconsin, United States). The following densitometric parameters were recorded: BMD, T-score, and Z-score. The T-score represented the difference between the obtained BMD measurement and the mean BMD for young adults, divided by the standard deviation for young adults. The Z-score was calculated as the difference between the measured BMD and the age-adjusted mean BMD divided by the standard deviation in the general population.

The T-score includes a comparison to “peak” bone mass, and indicates how many standard deviations the patient’s bone density differs from the average bone density of a healthy, young adult (approximately 20–30 years old) of the same sex. The T-score values employed in the current study were as follows: T-score ≥−1.0 was considered normal, T-scores between −1.0 and −2.5 were classified as osteopenia (reduced bone density), and T-scores ≤−2.5 were recognized as osteoporosis. The T-score is the basic indicator used to diagnose osteoporosis, and it was used to classify patients into healthy and osteopenic groups. The Z-score, in turn, provides a comparison to individuals of the same age. The Z-score indicates how many standard deviations the patient’s bone density differs from the average for individuals of the same age and sex. A Z-score ≥−2.0 indicates bone density appropriate for age. A Z-score <−2.0 shows bone density lower than expected for a given age. Nonetheless, the Z-score cannot be used to diagnose osteoporosis or osteopenia; it only indicates whether bone mass is normal or low. According to the guidelines applied for other autoimmune diseases, such as inflammatory bowel disease, the T-score is recommended for diagnosing secondary osteoporosis (Harbord et al., 2016).

### Handgrip strength

In all patients participating in the study, hand grip muscle strength was measured using a hydraulic dynamometer Baseline Hydraulic Hand Dynamometer. The highest score from the three measurements obtained with the dominant hand was considered the correct result, in accordance with the manufacturer’s recommendations.

### Laboratory tests

Laboratory parameters were marked in the central laboratory of the University Clinical Hospital in Poznan. Serum 25(OH)D concentration, total and ionized calcium, phosphates, and magnesium levels were assessed using the electrochemiluminescence binding method test and the Cobas e 601 analyzers (Roche, Basel, Switzerland).

### Vitamin D supplementation

Patients in the study group supplemented vitamin D in standard doses – 2000 IU/day, as recommended by the Polish Diabetes Association (2023) – as did the healthy individuals. Clinical recommendations for the management of people with diabetes 2023. Position of the Polish Diabetes Association (Anonymous, 2023, brak daty). According to the general recommendations, the supplementation period spanned from October to April.

### Clinical survey

All participants completed the original questionnaire referring to the supplementation of vitamin D, the presence of back pain and spontaneous fractures. The patients with diabetes also provided data regarding the duration of diabetes, the daily dose of insulin and the presence of chronic complications.

### Statistical analysis

Continuous variables were presented as medians and interquartile ranges Me [Q1–Q3], since data did not follow the normal distribution (Shapiro-Wilk test). The categorical data were presented as numbers and relative percentages. The interval data were compared by the Mann-Whitney test. Categorical data were analyzed by chi-square test of independence. The statistical analysis was performed using the statistical package Statistica 13.3 -TIBCO Software Inc. (2017). Statistica (data analysis software system), version 13. http://statistica.io.

The occurrence of osteoporosis or osteopenia was assumed to be approximately 20% in the study group and 0% in the control group. With this difference, the effect size assuming α = 0.05 and 80% power, the estimated n per group was n = 35 individuals per group, hence, a total of 70 participants were considered significant, at p < 0.05.

We assumed BMD (total body BMD) to be 10% lower than in the control group. In the control group, we observed BMD mean = 1.25, SD = 0.14. With these assumptions for the control group, we estimated a mean for the analyzed group to be lower by 10%, and assumed the same measure of data dispersion in the study group, as in the control group, i.e., a mean = 1.125 and SD = 0.14. Considering α = 0.05 and power 80%, we obtained n = 21 for the group, i.e., a total of 42 subjects. It was estimated that the decrease in vitamin D in the study group would be 30% (15.08/22.65 = 0.69). Considering α = 0.05 and power of 80, we obtained n = 28 for the group, i.e., 56 individuals in total. The results obtained from 66 patients with dm1 are sufficient to conduct the study.

## Results

The median age of patients included in the study was 38.7 years in the study group, and 38.5 years in the control group. The average duration of diabetes was 17 years, the patients did not present with chronic complications of diabetes. The median value of glycated hemoglobin amounted to 7.9% in the group with diabetes. Detailed characteristics of the study group are presented in Table 1.

**TABLE 1:** Characteristics of the study group at diagnosis. Median values and interquartile range (Q1–Q3) or value and percentage are provided.

Variable	DM1 (n = 66)		CG (n = 66)		p < 0.05
	Median value	[Q_1_–Q_3_]	Median value	[Q_1_–Q_3_]	
Age, y	38.7	32.4–45.1	38.5	28.5–43.5	0.41
Sex, female/male, n (%)	32(49%)/34 (51%)		45 (68%)/21(32%)		0.021
Body weight, kg	74.4	63.0–88.0	68.0	60.5–79.0	0.13
BMI, kg/m^2^	24.7	21.6–27.6	23.00	20.80–26.40	0.11
WHR, n	0.88	0.8–0.9	0.84	0.8–0.9	0.11
Diabetes duration, y	17	9.0–23.0	-	-	-
DDI, u/kg/day	0.57	0.49–0.64	-	-	-
HbA1c, %	7.9	7.0–8.5	-	-	-
FPG, mg/dL	140.5	86.5–181.0	88	84.0–94.0	-
Creatynin, μmol/L	76.9	65.4–84.9	69.8	60.9–81.3	0.22
25(OH)D, ng/mL	27.5	21.0 41.0	30.5	22.0–36.0	0.87
TSH, mlU/L	1.6	1.08–2.93	1.6	1.12–2.16	0.28
Smokers, %	14,55%		8.93%		0.38

Abbreviations: 25(OH)D, 25-hydroxyvitamin D; DDI, daily insulin dose; HbA1c, glycated hemoglobin; FPG, fasting plasma glucose; DM1, type 1 diabetes; CG, control group; TSH, thyroid-stimulating hormone.

On the basis of the performed densitometry, significant differences in bone mineral density were found in diabetic patients compared to young healthy adults matched for age and sex. These differences were observed both in the femoral neck area and in the lumbar spine. Osteoporosis (t- score) was not observed in any person from the study group. Osteopenia, based on the assessment of the femoral neck (t- score), was diagnosed in 12 patients with diabetes, and according to the assessment of the density of the lumbar spine (t- score) in 12 participants with DM1. Detailed data are presented in Table 2. In the diabetic study group, the Z-score indicated osteopenia in 12 individuals in FN (z-score), and in 0 patients with osteoporosis. L1-L4/Z-score in 12 subjects showed values typical for osteopenia.

**TABLE 2:** Densitometry tests results–a comparison of the study group and the control group. The Mann-Whitney U Test Results are presented as median values and interquartile range (Q1–Q3), p < 0.05 is statistically significant.

Variable	DM1 (n = 66)		CG (n = 66)		p < 0.05
	Median value	[Q_1_–Q_3_]	Median value	[Q_1_–Q_3_]	
BMD (FN), g/cm^2^	1.039	0.95 to 1.12	1.101	1.03 to 1.19	0.001
t-score (FN)	−0.1	−0.80 to 0.40	0.40	0.00 to 1.10	0.000
Z-score (FN)	0.0	−0.50 to 0.70	0.60	0.20 to 1.30	0.000
BMD (L1-L4)	1.203	1.12 to 1.29	1.221	1.17 to 1.33	0.084
T-score (L1-L4)	0.0	−0.80 to 0.80	0.25	−0.30 to 1.25	0.033
Z-score (L1-L4)	−0.1	−0.60 to 0.80	0.35	−0.20 to 1.00	0.027

Abbreviations: BMD, bone mineral density; DM1, type 1 diabetes; CG, control group; FN, femoral neck; L1 L4, Lumbar spine (L1–L4).

The group of patients with and without diabetes also differed in terms of the content of phosphates and magnesium in the blood. Concentrations of magnesium and phosphates were significantly lower in the group with diabetes, yet they remained within the normal range. The group with diabetes and without diabetes did not differ in terms of vitamin D levels and total and ionized calcium (Table 3).

**TABLE 3:** Laboratory tests. Results are presented as median values and interquartile range (Q1–Q3), the Mann-Whitney U Test, p < 0.05 is statistically significant.

Variable	DM1 (n = 66)		CG (n = 66)		p < 0.05
	Median value	[Q_1_–Q_3_]	Median value	[Q_1_–Q_3_]	
25(OH)D,	27.50	21.00–41.00	28.00	22.00–36.00	0.870
Ionized calcium	5.24	5.12–5.32	5.28	5.15–5.43	0.081
Total calcium	9.66	9.44–9.91	9.48	8.98–9.91	0.053
Inorganic Phosphorus	3.50	3.20–3.87	4.66	3.52–4.35	0.005
Magnesium	1.91	1.81–2.02	2.17	2.05–2.44	<0.0001

Abbreviations: DM1, type 1 diabetes; CG, control group.

The study group and the control group were asked to complete a proprietary questionnaire regarding the symptoms associated with the occurrence of osteoporosis and osteopenia. According to the survey, it was demonstrated that diabetic patients significantly more often suffered from pain in the lumbar spine unrelated to radiculopathy, as well as observed a considerable weakening of muscle strength during compression. Furthermore, diabetic patients significantly more often reported fractures as a result of trauma than controls (see Table 4 for details).

**TABLE 4:** Results obtained from original questionnaire referring to the supplementation of vitamin D and the symptoms of osteoporosis using Pearson Chi-square test. The results are presented as values and percentages, P < 0.05 is statistically significant.

Variable	DM1	CG	p < 0.05
Fractures due to trauma n/(%)	14 (27.45)	1 (2.13)	0.000
Idiopathic fractures n/(%)	0 (0)	0 (0)	-
Low back pain not related to radiculopathy occurring at least once a month (%)	21 (43.75)	2 (4.26)	0.0001
Muscle weakness observed by the patient while performing a handshake n/(%)	23 (47.92)	1 (2.13)	0.0001

Abbreviations: DM1, type 1 diabetes; CG, control group.

In the conducted study, no differences were observed between the groups with diabetes and osteopenia, as well as those without osteopenia in relation to the duration of diabetes. Diabetes duration in patients with type 1 diabetes with normal BMD (FN) was 15 (7–23) years vs. 15 (10–20) years, p = 0.517 in BMD osteopenia (FN). No changes were also found in (L1-L4) – diabetes duration time was 14.5 (8–23) years in patients with DM 1 with normal BMD (L1-L4) vs. 18 (12–24) years, p = 0.554 in DM 1 patients with BMD osteopenia (L1-L4).

Moreover, the analysis of vitamin D concentrations in the study and control groups was also performed. Severe vitamin D deficiency was found in 1 person with osteopenia of the femoral neck and in 2 participants with osteopenia in the L1-L4 segment of the spine. Only 50% of subjects with type 1 diabetes and osteopenia received the recommended level of vitamin D (Table 5).

**TABLE 5:** Vitamin D level in the study groups Results are presented as values and percentages, P < 0.05 is statistically significant.

Category	DM 1 normal BMD (FN)	DM 1 BMD osteopenia (FN)	GC (FN)	P < 0.05	DM 1 normal BMD (L1-L4)	DM 1 BMD osteopenia (L1-L4)	GC (L1-L4)	P < 0.05
Severe deficiency (0–10) n.%	3 (6)	1 (13)	0 (0)	p = 0.214	2 (5)	2 (14)	0 (0)	p = 0.122
Deficiency (10–20) n.%	8 (16)	2 (25)	10 (17)		8 (18)	2 (14)	10 (17)	
Suboptimal concentration (20–30) n.%	17 (34)	1 (13)	29 (50)		15 (34)	3 (21)	29 (50)	
Optimal concentration (30–80) n.%	21 (42)	4 (50)	19 (33)		18 (41)	7 (50)	19 (43)	
Toxic concentration (>100) n.%	1 (2)	0 (0)	0 (0)		1 (2)	0 (0)	0 (0)	

Abbreviations: DM1, type 1 diabetes; CG, control group; BMD, bone mineral density; FN, femoral neck.

There were also no differences in bone mineral density depending on vitamin D supplementation in participants with diabetes and the control group (Table 6).

**TABLE 6:** A comparison of vitamin D concentration, bone mineral density, the T-score and Z-score of the lumbar spine (L1–L4) and femoral neck in patients with type 1 diabetes with and without vitamin D supplementation. The Mann-Whitney U test results are presented as median values and interquartile range (Q1-Q3).

Variable	DM 1 Vitamin D supply (n = 25)	DM 1 Vitamin D non supply (n = 41)	p < 0.05	GC Vitamin D supply (n = 13)	GC vitamin D non supply (n = 53)	p-value
	Me [Q_1_–Q_3_]	Me [Q_1_–Q_3_]		Me [Q_1_–Q_3_]	Me [Q_1_–Q_3_]	
25(OH)D,	41 [27 to 46]	22 [17 to 30]	<0.0001	43 [35 to 49]	25.5 [21 to 29.5]	<0.0001
BMD (FN), g/cm^2^	1.07 [0.99 to 1.12]	1.02 [0.94 to 1.1]	0.335	1.09 [1.04 to 1.1]	1.1 [1.03 to 1.19]	0.459
t-score (FN)	0.1 [−0.3 to 0.6]	−0.15 [−0.85 to 0.3]	0.227	0.2 [−0.1 to 0.5]	0.5 [0 to 1.1]	0.336
Z-score (FN)	0.3 [-0.2 to 1.1]	−0.1 [−0.55 to 0.65]	0.206	0.5 [0.2 to 1]	0.65 [0.2 to 1.3]	0.898
BMD (L1-L4)	1.04 [0.97 to 1.1]	1.02 [0.94 to 1.11]	0.809	1.07 [0.98 to 1.12]	1.07 [0.99 to 1.15]	0.641
T-score (L1-L4)	0 [−0.9 to 0.5]	−0.35 [−1 to 0.2]	0.233	0.2 [−0.6 to 0.6]	0.1 [−0.2 to 0.9]	0.552
Z-score (L1-L4)	−0.1 [−0.8 to 0.7]	−0.2 [−0.8 to 0.3]	0.307	0.4 [−0.1 to 1.1]	0.3 [−0.1 to 0.8]	0.993

Abbreviations: BMD, bone mineral density; DM1, type 1 diabetes; CG, control group; F, femoral neck; L1-L4, Lumbar spine (L1–L4).

## Discussion

Although the interdependence of bone and hyperglycemia remains unclear, it is likely that glycaemia may affect bone metabolism differently. The underlying pathomechanism is complex and depends on the type of diabetes, as well as on the stage of the disease. There are several risk factors associated with diabetes, which affect bone metabolism; the first is hyperglycemia. Increased glycaemia may affect bone cells directly, and in turn, substances present in bone cells may influence glucose metabolism. Furthermore, the development of complications in diabetic patients can also affect the bone tissue. Due to an early onset and long duration of the disease, as well as the lack of any secretory function of the pancreas, patients with type 1 diabetes are at a higher risk of this complication, which was confirmed by our study. In the group of participants with type 1 diabetes, compared to the controls of similar age, a considerably higher incidence of osteopenia was observed. The patients participating in our study were randomly recruited and did not report any prior symptoms from the osteoarticular system. None of the diabetic patients had been diagnosed with osteoporosis, however, the incidence of osteopenia was significantly higher when assessing both the lumbar and femoral neck. The Vestergaard study showed that both type 1 and type 2 diabetes showed an increased risk of bone fractures. In contrast, the BMD Z-score in the patients they analyzed was significantly lower in patients with type 1 diabetes than in patients with type 2 diabetes. In patients with type 2 diabetes, a higher BMI is probably protective, and therefore such a reduction in bone mass was not observed (Vestergaard, 2007). Similar results were also obtained in other studies in patients with type 1 diabetes (Eller-Vainicher et al., 2011). At study by Hamilton et al., bone mineral density was prospectively assessed in 26 patients with type 1 diabetes. After a 5-year study follow-up, a decrease in mineral density in the femoral neck area was found in 17 men, but no changes were observed in the lumbar spine and forearm. However, there was no BMD reduction in women with type 1 diabetes (Hamilton et al., 2012; Maser et al., 2012). The age at which diabetes is diagnosed also affects the occurrence of osteoporosis or osteopenia. Studies conducted on adolescents with type 1 diabetes indicated that high values of glycated hemoglobin and poor glycemic control may cause growth retardation and reduction of bone mineral density in adolescents with diabetes (Hamed et al., 2011).

Most complications of diabetes develop after many years of disease, therefore the duration of diabetes is reported as a significant risk factor for the development of osteoporosis and osteopenia (Bilha et al., 2021). In our group, the median duration of diabetes was 17 years. Despite the relatively long duration of the disease, the analysis did not demonstrate that the duration of diabetes significantly influenced the development of osteopenia. This may be associated with earlier better control of diabetes or other factors, such as genetics, that predispose to the development of osteopenia and osteoporosis.

It is of note that there is a variety of risk factors for bone mineral disorders in T1DM patients. One of the risk factors for the development of bone mineral disorders is oxidative stress, which is characteristic for type 1 diabetes mellitus. Oxidative stress-induced cytokines and reactive oxygen species exert a major impact on the formation and survival of osteoclasts, osteoblasts and osteocytes. Furthermore, oxidative defense by FoxO transcription factors is essential for maintaining skeletal homeostasis. The increased accumulation of advanced glycation end products (AGEs) in diabetic patients also affects bone metabolism. Interestingly, the receptor for AGEs (RAGE) is also a receptor for the high-mobility group box 1 (HMGB1) protein, which plays a crucial role in osteoclastogenesis. HMGB1, in turn, is released from macrophages in response to the stimulation with the osteoclast differentiation factor RANKL (receptor activator for nuclear factor κ B ligand). Additionally, in patients with type 1 diabetes using insulin doses over their requirements, insulin resistance and hyperinsulinemia may also affect bone metabolism.

In most cases, however, the catabolic state induced by insulin deficiency reduces the formation of bone matrix proteins and reduces bone mineralization. The occurrence of ketoacidosis in poorly balanced patients with type 1 diabetes causes an increase in osteoclast activity.

It is worth bearing in mind that the occurrence of complications may further exacerbate bone disorders. In our group, we did not include individuals with GFR <60 and diabetic kidney disease, in which disorders of 1,25-dihydroxycholecalciferol production and disorders of calcium and phosphate metabolism may significantly affect the occurrence of bone complications. Moreover, diabetic angiopathy disorders also affect the microvascular blood flow and may impair the nutrition of bone tissue.

In addition, frequent malnutrition occurring in patients with type 1 diabetes may also intensify disorders of calcium and phosphate metabolism. In a study by Brown et al., subjects with type 1 diabetes showed higher calcium and magnesium excretion levels than individuals without diabetes. Hence, the association was observed between the excretion of both calcium and magnesium and HbA1. The higher the glycated hemoglobin index, the greater was the excretion of calcium and magnesium, and thus the greater the risk of complications related to chronic loss of calcium and magnesium (Brown et al., 1999). In our study, we measured both total and ionized calcium levels. This is even more significant in patients with chronic diseases, such as diabetes. The level of ionized calcium (not bound to proteins) may increase under stress, and may cause the activation of enzymes and mediate the response of cells to hormones. In our study, there were no differences in the values of total and ionized calcium between the two groups. However, there were differences in the values of magnesium and phosphate in the serum. Diabetic subjects showed lower phosphate and magnesium concentrations than non-diabetic participants, although these values were still within the normal range. The Ching-Chiang Lin study conducted on patients with type 1 diabetes also demonstrated significantly lower magnesium concentrations in patients with type 1 diabetes than in the control group. Moreover, these values were found to be considerably lower in individuals with poor glycemic control (Lin and Huang, 2015). Similar results were obtained in the study by Inácio (Inácio et al., 2022). Nevertheless, other studies suggested a relationship between low serum magnesium levels, glycemic control (HbA 1c) and C-reactive protein levels in T1DM patients and features of insulin resistance (using high doses of insulin) (Oost et al., 2022). Phosphates may also play an important role in the process of bone demineralization through a close relationship with cellular glucose metabolism in the production of energy. In addition, in the presence of complications of diabetes, ectopic deposits of calcium phosphate are formed, additionally affecting bone metabolism. In the study by Bilha, however, no differences were observed in the phosphate values in the DM1 and the control groups in the terms of changes in bone mineral metabolism (Bilha et al., 2021). Conversely, our study found significantly lower phosphate levels in the DM1 group.

One of the well-documented and widely established risk factors for bone mineral disorders is vitamin D deficiency. In fact, various conducted studies confirmed a significant relationship between vitamin D deficiency and the occurrence of type 1 diabetes (Pozzilli et al., 2005; Littorin et al., 2006; Cooper et al., 2011). In addition, vitamin D deficiency also correlates with poorer diabetes control and the risk of complications, such as diabetic retinopathy and nephropathy (Kaur et al., 2011). The major genes that control vitamin D levels were strongly linked with autoimmune diseases, such as T1DM. The gene, CYP27B1, encoding the 1-α-hydroxylase that activates the last step in the synthesis of vitamin 1,25D3, was identified as associated with the susceptibility to T1DM (Bailey et al., 2007). Therefore, the connection of this vitamin with bone metabolism in individuals with type 1 diabetes becomes indispensable. The role of vitamin D in the treatment of patients with diabetes is also emphasized in the literature. A study of 70 T1DM patients revealed a lower insulin dose after 3 and 6 months of vitamin D supplementation (Pitocco et al., 2006). However, other studies did not observe a similarly beneficial effect (Li et al., 2009; Ataie-Jafari et al., 2013; Ludvigsson et al., 2021). According to the study conducted by Nwosu, a smaller increase in HbA1c was found in subjects supplementing vitamin D than in the participants in the control group (Nwosu et al., 2022). Furthermore, it was shown that hypovitaminosis D may lead to the development of neuropathic changes in the early stages of diabetes, particularly in the nerves of the lower limbs (Polat et al., 2022). Due to the association of low levels of vitamin D with the risk of developing DM 1, various attempts were made to verify whether vitamin D supplementation may delay the process of pancreatic islet loss and improve glycemic control at an early stage of the disease. Nevertheless, the results of these studies were not satisfactory. Shih et al. found no significant reduction in insulin requirements following 6 months of treatment with cholecalciferol at a dose of 25,000 IU/week. In a randomized study in children with T1D and trace pancreatic function (measurable C-peptide >0.2 pmol/mL), substitution with cholecalciferol (2000 IU/day) did not prevent further loss of pancreatic secretory function (Bizzarri et al., 2010; Walter et al., 2010; Shih et al., 2016). Conversely, when evaluating the effect of vitamin D in the management of diabetic complications, paricalcitol was shown to significantly reduce the rate of urinary albumin excretion compared to placebo in T1D patients with diabetic nephropathy, although no effect on cardiovascular risk markers was observed after 12 weeks of treatment (Joergensen et al., 2015). However, up to date there have been no studies with regard to vitamin D supplementation as a means of osteoporosis prevention in T1DM. Clinical trials conducted on other groups of patients confirm this relationship. It should be noted that significantly lower vitamin D levels were also observed in children newly diagnosed with type 1 diabetes. In addition, less exposure to sunlight in childhood was associated with a higher risk of developing type 1 diabetes. Another study assessed melanin levels in patients with type 1 diabetes, and found a loss of melanocytes in T1DM patients. Notably, melanin content in the skin was associated with glycemic control in type 1 diabetes. Lower melanin content indicated a higher risk of developing microvascular complications of diabetes (Mackiewicz-Wysocka et al., 2014).

Vitamin D and calcium supplementation was found to reduce the risk of total fractures by 15% and reduces the risk of hip fractures by 30% in healthy individuals (Weaver et al., 2016). Similar studies showed the effect of vitamin D supplementation on bone mineral density in post-menopausal women (Liu et al., 2020). The NHANES III research also confirmed the relationship between serum 25(OH)D concentration and hip BMD in younger subjects (20–49 years) (Bischoff-Ferrari et al., 2004). Moreover, the role of vitamin D was also discussed in other autoimmune diseases, such as inflammatory bowel disease (IBD). The studies emphasized the role of vitamin D deficiency in the development of osteoporosis in patients with IBD and, as with diabetes, vitamin D deficiency was also identified as a factor in the development of the abovementioned immunological diseases (Szymczak-Tomczak et al., 2022). However, research involving vitamin D supplementation in patients with inflammatory bowel diseases did not yield such promising results. For instance, Bernstein et al. evaluated the effect of calcium and vitamin D on the treatment of osteoporotic changes in patients with IBD. Although supplementation slightly improved the condition of the lumbar spine (BMD), this effect was not statistically significant (Bernstein et al., 1996). The major studies conducted with regard to vitamin D supplementation in diabetes were related to preventing the development of type 2 diabetes and reducing insulin resistance, thus, preventing the development of type 1 diabetes, reducing the symptoms of complications, such as peripheral neuropathy, and preventing comorbidities, e.g., cardiovascular diseases. The effect of vitamin D on reducing osteoporosis in individuals with diabetes has not been studied. In our study, no differences were found in bone mineral density between participants supplementing vitamin D and those not receiving vitamin D, both in the control group and in the diabetic group. However, we observed that only 50% of subjects with diabetes and osteopenia showed normal levels of vitamin D. Interestingly, the optimal level of vitamin D in patients with diabetes without osteopenia had only 40% of people.

It is vital to bear in mind that in patients with diabetes an increased risk of bone fractures is reported, due to an elevated incidence of falls during hypoglycemia and microvascular disease, occurring particularly in long-standing type 1 diabetes (Oei et al., 2013). Numerous studies also demonstrated an increased risk of falls in diabetic patients.

In view of all the aforementioned data, it is evident that the prevention of osteoporosis and the maintenance of normal bone mass is vital for T1DM patients. Standardized screening with regard to bone mineral density (densitometry) should be included in the management guidelines for individuals with diabetes. In addition, preventing vitamin D deficiency and maintaining a healthy calcium and phosphate balance may positively affect bone health in patients with type 1 diabetes.

Although we were deeply committed to ensure the quality of our study, it bears certain limitations. Firstly, the study group was relatively small. Secondly, we did not consider the dose of vitamin D. Although in Poland, vitamin D is available as a medication or a dietary supplement, its pharmacokinetics are not well known. Therefore, we did not include this data in our analysis. Additionally, many patients did not know their vitamin D dose. Finally, we did not include information regarding the season in which the 25OHD tests were performed. On the other hand, the time spent outdoors represents a more relevant variable in terms of sun exposure. Thus, it could have resulted in the creation of further subgroups and decrease the quality of the statistical analysis.

## Conclusion

1. In patients with type 1 diabetes, bone mineral density in the area of the femoral neck and lumbar spine is significantly reduced in comparison to healthy individuals.
2. The lack of differences in the values of total and ionized calcium between the study groups indicates a lack of early and simple markers to screen for bone mineral metabolism disorders in T1DM patients.
3. The difference in the concentration of magnesium and inorganic phosphorus between the groups with and without diabetes requires further research, which would focus on the impact of hyperglycemia on bone mineral metabolism.
4. The presented study did not demonstrate that the duration of diabetes may affect the development of osteopenia in individuals with diabetes.
5. There were no differences in the level of vitamin D between the study groups. There were also no differences in bone mineral density between participants supplementing vitamin D and who did not receive vitamin D supplements, both in the control group and in the group of diabetics.

## Data Availability

The original contributions presented in the study are included in the article/supplementary material, further inquiries can be directed to the corresponding authors.
